# Fall From a Car Resulting in a Right Upper Limb Axillary Artery Vascular Injury

**DOI:** 10.7759/cureus.79520

**Published:** 2025-02-23

**Authors:** Mashhood U Qazi, Rouda R AlEissaee, Hind S Mubarak Mohamed Al Dhaheri, Soheir A Mohammed Aboelmaaty

**Affiliations:** 1 Emergency Department, Tawam Hospital and Sheikh Tahnoon Bin Mohammed Medical City, Al Ain, ARE; 2 Emergency Department, Sheikh Tahnoon Bin Mohammed Medical City, Al Ain, ARE

**Keywords:** axillary artery injury, humerus fracture, skin graft, subclavian artery, trauma

## Abstract

A young male patient was brought to the Emergency Department after falling from a moving car. It was noted that he had a left scalp hematoma with a deformed right upper limb. Computer tomography of the head, cervical spine, thoracic and lumbar spine, thorax, abdomen, and pelvis was performed. The CT scan showed multiple cervical spine fractures, a fracture of the first rib with localized pneumothorax, and multiple lung contusions. There was also a fracture of the right humerus. CT angiography showed evidence of a complete cutoff of the right axillary artery at its origin in the axilla, which was compressed by a hematoma. He was immediately taken for emergency surgery by the trauma team and underwent right subclavian artery grafting by the vascular surgical team. The plastic surgery team performed a right forearm fasciotomy and right brachial plexus repair. The orthopedic team applied an external fixator to the right humerus. During his hospital stay, the patient developed severe sepsis presumed to be due to pneumonia, and was treated with piperacillin-tazobactam along with vancomycin. He was discharged after a hospital stay to a long-term facility for continued physiotherapy.

## Introduction

Axillary artery injuries can result from direct trauma as well as trauma to nearby structures. Axillary artery injuries have been known to be associated with scapulothoracic dissociation usually accompanied by brachial plexus and axillo-subclavian vessel injuries [1]. Shoulder dislocations can result in axillary artery injuries but are rare [2]. However, traumatic axillary artery injuries have been scarcely reported in medical literature. Boudreau et al. identified patients with traumatic axillary and subclavian arterial injuries over a period of six years in which blunt and penetrating trauma were equally represented [3]. Another study by Angus et al. identified a total of 221 patients with isolated axillary artery injuries over a five-year period [4]. Iatrogenic axillary artery injuries can also occur. In a study by Umeda, an axillary artery injury occurred during the resection of a chest wall tumor [5]. Proximal humerus fractures have also been associated with axillary artery injuries. The fracture of the upper end of the humerus with axillary artery injury is rare with limited literature available [6,7]. Menendez identified 331 patients with axillary artery injuries out of 388,676 patients who had a proximal humerus fracture [8].

In this case report, we present a case of a young patient who accidentally fell from a moving car and sustained a complete cutoff of the right axillary artery. Such a case has hardly been reported in the literature in the past.

## Case presentation

A 40-year-old male patient was brought to the Emergency Department with a history of a fall from a moving car. His initial vital signs were stable with a blood pressure of 114/68 mmHg, respiratory rate of 18 breaths per minute, oxygen saturation of 98% on room air, and heart rate of 90 per minute. He had a Glasgow Coma Score (GCS) of 14/15 with a motor response of 6/6, verbal response of 4/5 (due to confusion), and eye response of 4/4. On physical examination, the positive findings were a left scalp hematoma, reduced air entry on the right side of the chest with normal chest movements, and no external wounds or bruising of the chest. In addition, the patient also had a deformed mid-right upper limb with non-palpable right distal upper limb pulsations. The rest of the physical examination was unremarkable.

**Figure 1:**
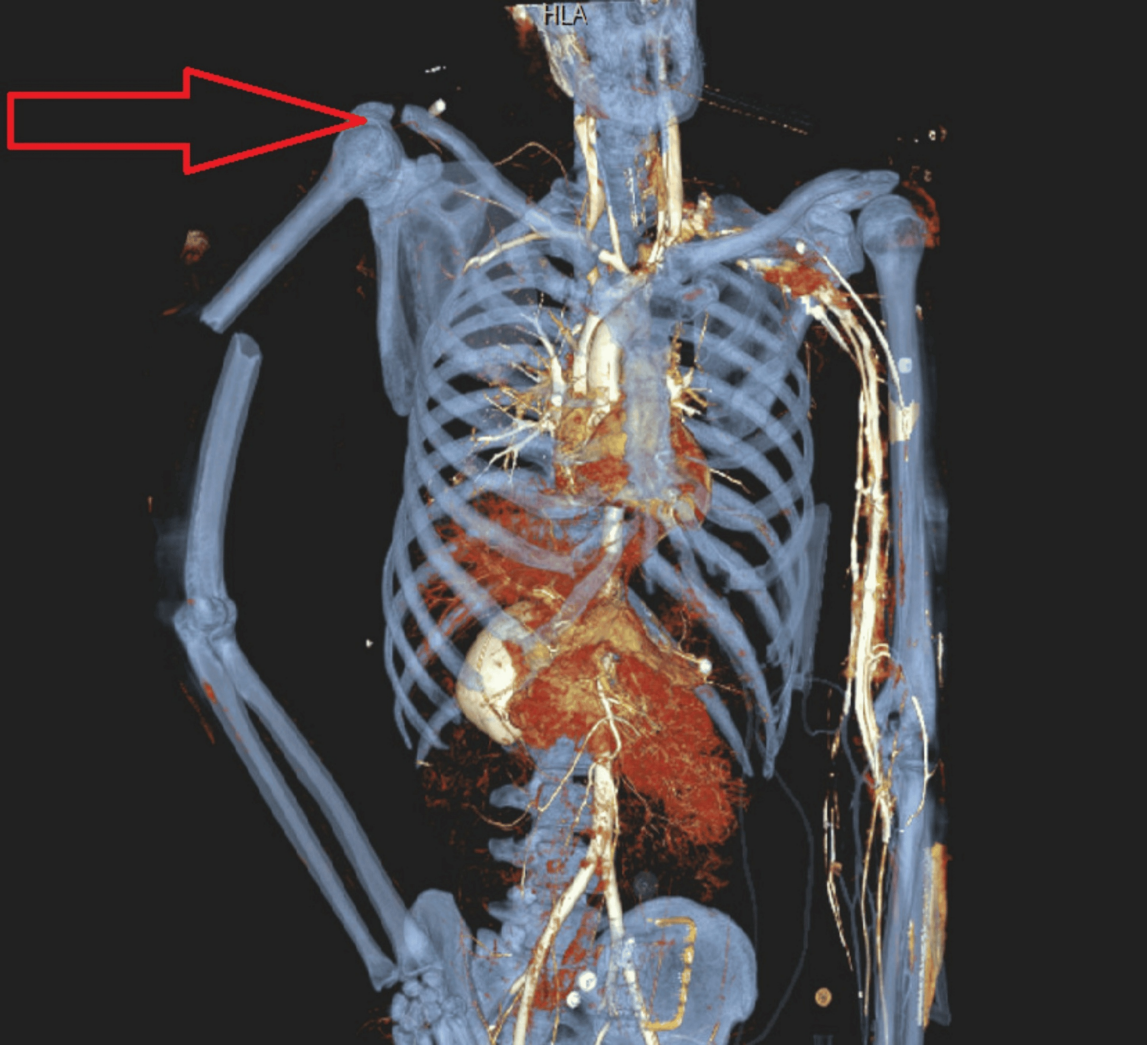
3-D reconstruction view demonstrating a right humerus fracture and lack of vascular supply in the right upper limb The red arrow points to the right upper limb. It shows that there is a lack of contrast in right upper limb blood vessels as well as a fracture of the shaft of the right humerus.

**Figure 2:**
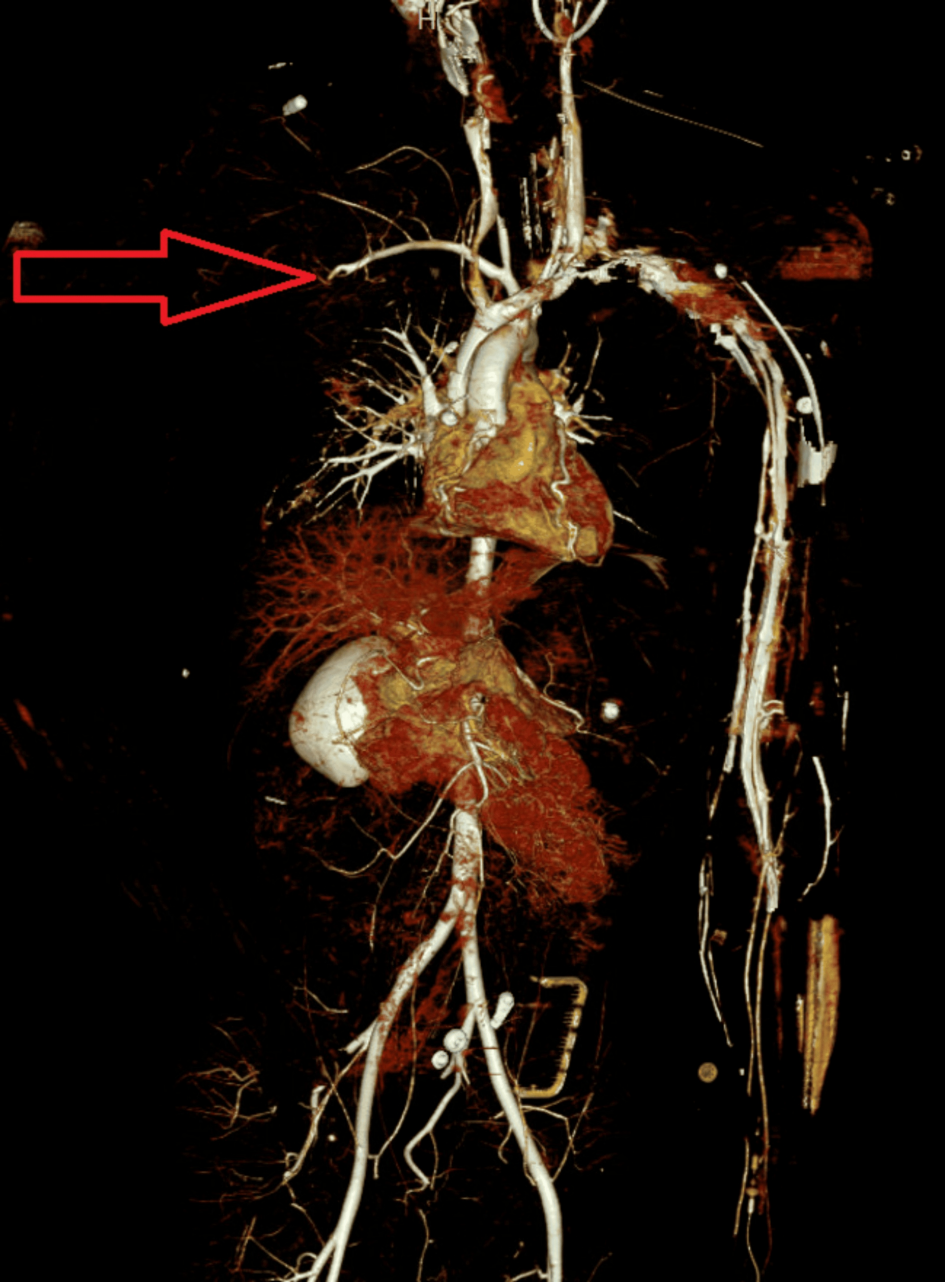
CT angiogram view demonstrating lack of blood supply in the right upper limb The red arrow demonstrates that there is lack of blood supply in the right upper limb as compared to the left upper limb.

**Figure 3:**
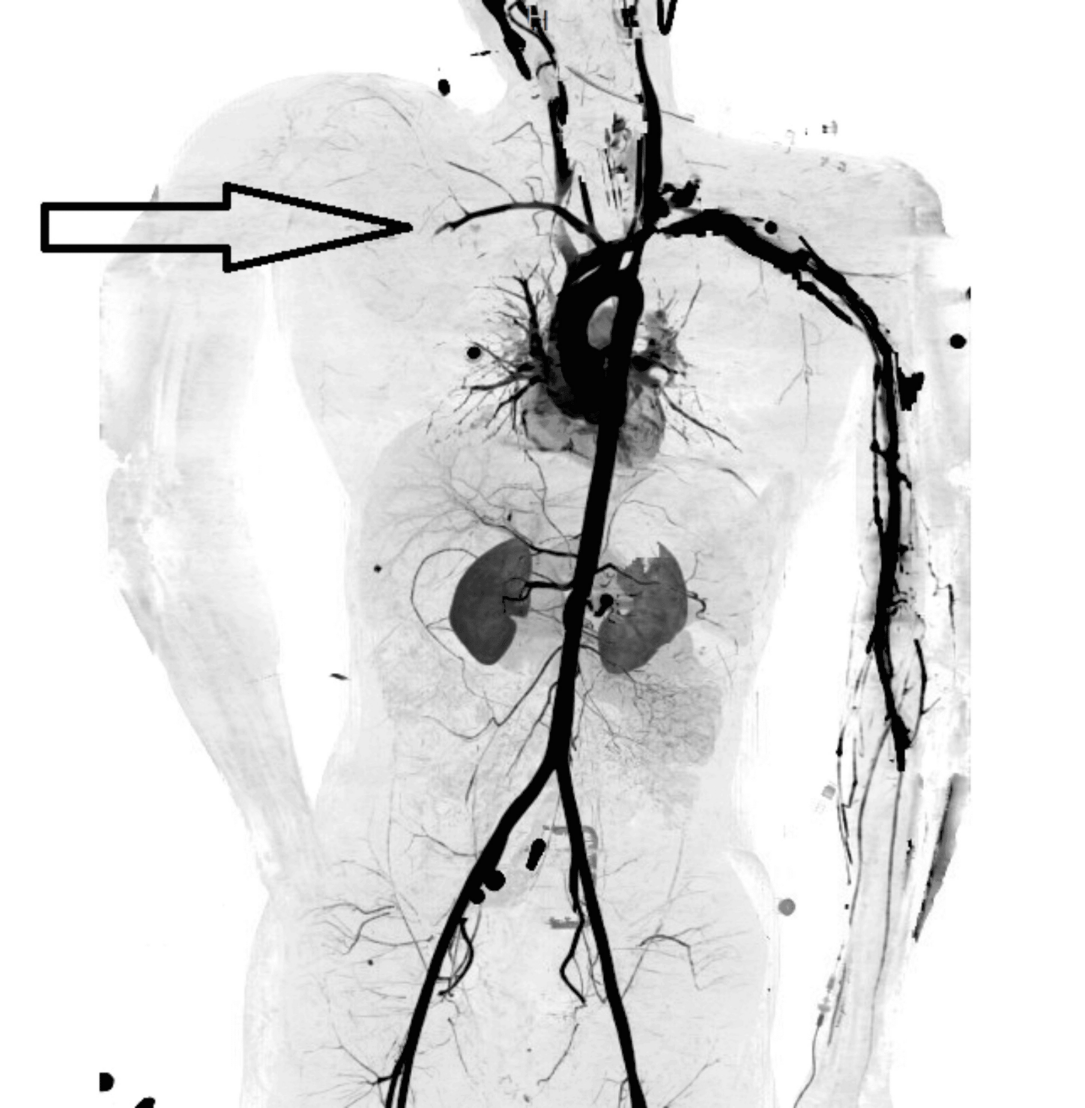
CT 3-D reconstruction view demonstrating no blood supply in the right upper limb The black arrow points to the lack of blood supply in the right upper limb blood vessels compared to the left upper limb.

As per the hospital trauma protocol, blood investigations were requested, including full blood count, urea and electrolytes, clotting profile, venous blood gas, and blood cross-match, and the patient was given intravenous analgesia, including intravenous paracetamol and morphine. Computer tomography (CT) revealed multiple findings. CT of the cervical spine revealed an un-displaced fracture of the anterior arch of the first cervical spine vertebra along with a fracture of the transverse process of the seventh cervical spine vertebra on the right. Thoracic CT showed a fracture of the first rib with minimal localized pneumothorax seen in the medial aspect of the lower right upper lobe. There were also multiple lung contusions. There was a displaced fracture of the mid-shaft of the right humerus. Due to the absence of pulsations in the distal right upper limb, a right upper limb CT angiogram was requested. CT angiography of the right upper limb showed that there was evidence of a complete cutoff of the right axillary artery at its origin in the axilla (Figures 1-3), which was compressed by a hematoma. There was an expanding hematoma in the lateral and posterior aspect of the chest wall with extension and expansion seen in the right axilla.

He was taken immediately for emergency surgery by the trauma team. He underwent right subclavian artery grafting by the vascular surgical team. The plastic surgery team performed right arm forearm fasciotomy and right brachial plexus repair. The orthopedic team applied an external fixator to the right humerus. Cervical spine fractures were treated conservatively with a soft neck collar by the neurosurgical team. The patient was subsequently admitted to the intensive care unit.

A week later, the patient underwent debridement of devitalized tissues of the right arm and fasciotomy for right forearm wounds, along with partial primary closure of the right arm and right forearm wounds performed with a skin graft taken from the right thigh.

**Figure 4:**
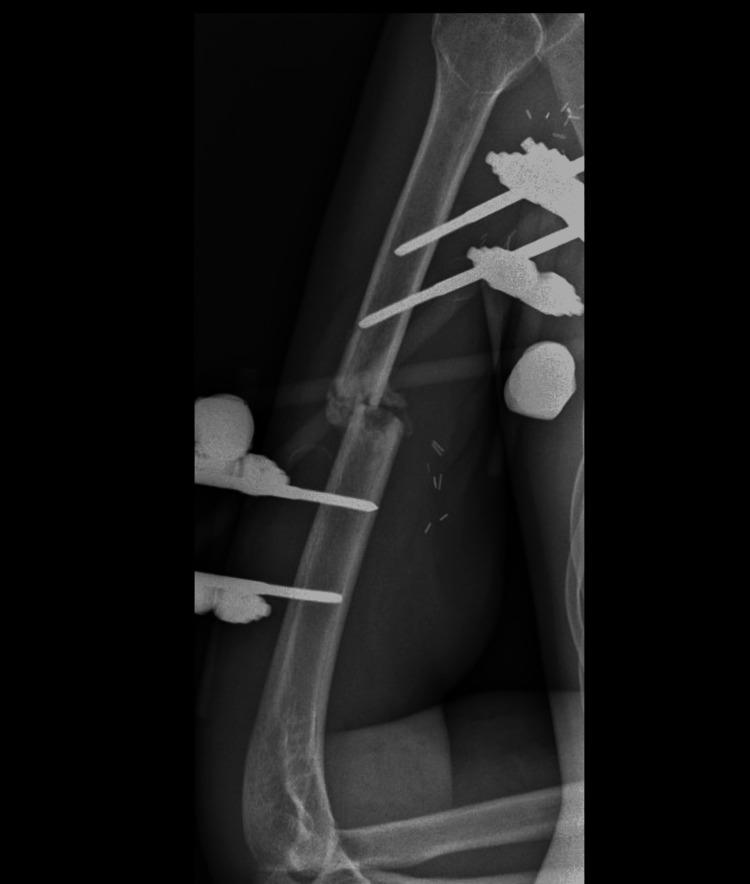
The three-month postoperative right humerus X-ray demonstrating external fixator application

He stayed in the intensive care unit for two weeks and was later admitted to the general surgical ward. During his two-month hospital stay, the patient developed severe sepsis, presumed to be due to hospital-acquired pneumonia (HAP), and was treated with piperacillin-tazobactam along with vancomycin. He was seen by the orthopedic team for a follow-up in two months, with satisfactory progress (Figure 4). He was discharged after two months to the long-term facility for continued physiotherapy to his right upper limb.

## Discussion

In a patient who has sustained traumatic distracting injuries, for example, injuries involving multiple systems, clinical identification of axillary artery transection could be extremely difficult. Pain, expanding hematoma, coolness, pallor, pulse deficit, and neurological dysfunction could be clinical signs of vascular injury and should not be ignored. Vascular injury signs and symptoms may appear either early or late [9]. The axillary artery is divided into three parts, and possible injuries could include rupture, occlusion, or pseudo aneurysm [10]. According to Ergünes et al., in the context of axillary artery injury after shoulder dislocation, the third part is the most commonly transacted portion of the axillary artery [11]. Khiami and Whittam have reported as high as 90% axillary artery injuries in the third portion. Injury to the axillary artery needs urgent vascular consultation and management [12,13]. There are different approaches for the treatment of axillary artery injuries and might include both open repair and endovascular repair. According to Angus et al., the majority of isolated axillary artery injuries were managed by open repair (82.8%) as compared to endovascular (10.2%) [4].

## Conclusions

In a poly-trauma patient with distracting injuries, a high index of suspicion should be present for vascular injury, especially when an expanding hematoma is present. In this case report, we presented a patient who had sustained an injury after a fall from a moving vehicle and had multiple distracting injuries including a head injury, chest injury, and right upper limb trauma. However, due to an expanding hematoma in the axillary region and suspected vascular injury, the patient underwent poly-trauma computer tomography, including computer tomography angiography, which revealed a right axillary artery injury.
